# Incidence and predictors of chronic kidney disease among patients with diabetes treated at governmental hospitals of Harari Region, eastern Ethiopia, 2022

**DOI:** 10.3389/fpubh.2023.1290554

**Published:** 2024-01-05

**Authors:** Abera Cheru, Dumessa Edessa, Lemma Demissie Regassa, Tesfaye Gobena

**Affiliations:** ^1^School of Environmental Health Science, College of Health and Medical Science, Haramaya University, Harar, Ethiopia; ^2^School of Pharmacy, College of Health and Medical Sciences, Haramaya University, Harar, Ethiopia; ^3^School of Public Health, College of Health and Medical Sciences, Haramaya University, Harar, Ethiopia

**Keywords:** chronic kidney disease, diabetes mellitus patients, incidence, predictors, eastern Ethiopia

## Abstract

**Background:**

Chronic kidney disease (CKD) is the leading cause of morbidity and mortality in diabetic patients. However, limited evidence is available about its incidence and predictors in Ethiopia, specifically in the Harari region.

**Methods:**

A retrospective follow-up study was conducted among 520 diabetes patients who followed their treatment at governmental hospitals in the Harari region between 1 September 2012, and 30 May 2022. The risk of developing CKD was calculated with a 95% CI, and the risk was stratified by type of diabetes mellitus. Predictors of CKD were determined using the Gompertz regression model with the baseline Cox model.

**Results:**

Data from 494 patients were included in the final analysis with 26 (5%) excluded. A total of 51 patients (10.32%) developed CKD over the 10-year follow-up period with an incidence rate of 2.16 cases (95% CI 1.64–2.84) per 100 person-years of observation. The risk of CKD was increased by three times (AHR: 3.09, 45 95% CI: 1.56, 6.14) among patients older than 60 years and by more than three times (AHR: 3.53, 95% CI: 1.43, 8.71) among patients with diabetes mellitus for longer than 5 years of stay with the diabetes mellitus. Moreover, the risk of CKD was increased four-fold among patients with high-density lipoprotein cholesterol (HDL-C) levels <40 mg/dL (AHR: 3.84, 95% CI, 1.80–8.18) and those with positive baseline proteinuria (AHR: 3.77, 95% CI: 1.43–8.71).

**Conclusion:**

We found that one in ten diabetic patients had developed CKD within 10 years of the diabetes mellitus diagnosis. Advanced age, longer duration of diabetes, lower baseline HDL-C level, and proteinuria had increased the hazards of developing CKD. We recommend a more focused follow-up of older adult patients with advanced disease status at baseline for optimal control of diabetes mellitus that prevents its furthering to CKD.

## Introduction

Chronic kidney disease (CKD) is defined as abnormalities of kidney structure or function, present for >3 months, with implications for health (1). It is a leading cause of morbidity and mortality in diabetic patients, representing a huge health and economic burden (2). CKD is divided into five stages based on levels of kidney function. Many authors now refer to moderate or clinically significant CKD as stages 3 and 4, with 60 mL/min chosen as a cutoff because it represents a loss of approximately 50% of the normal renal function value in young adult men and women, which is approximately 125 mL/min/1.73 m^2^ (3).

Globally, in 2017, there were 697.5 million cases of all-stage CKD, and 1.2 million people died each year due to the high economic cost of treatment (4). A Global Burden of Disease study in 2015 showed that over 2 million people died in 2010 because they had no access to dialysis (5). In addition, it has been estimated that, by the year 2030, approximately 2.3–7.1 million adults will have died prematurely from lack of access to renal replacement therapy (6). The number of deaths from CKD has increased; it was ranked 13th in 2016 and 12th in 2017, and forecasts indicate that by 2040, it will rank 5th globally in terms of years of life loss (4, 7, 8).

Globally, diabetes and hypertension are two common causes of CKD. In total, 30 (30%) to 50 (50%) of CKD and ESRD cases worldwide are caused by diabetes (9). The causes of CKD are heterogeneous in low- and middle-income countries. In sub-Saharan Africa, diabetes, hypertension, and HIV appear to account for only a portion of the significant CKD burden, especially in urban settings, where many risk factors remain undetermined (10, 11).

CKD is a major determinant of the poor health outcomes of non-communicable diseases. CKD is related to an 8–10-fold increase in cardiovascular mortality and is a risk multiplier in patients with diabetes and hypertension. CKD due to diabetes and hypertension affects 5–7% of the world’s population and is more common in developing countries (3).CKD among diabetic patients is associated with increased premature mortality, end-stage renal disease, the need for renal replacement therapy, cardiovascular diseases, and escalating healthcare costs (12). CKD is a worldwide public health issue, both in terms of patient population and treatment costs (13).

In Africa, the overall prevalence of CKD stages 1–5 is estimated to be 15.8%, with up to 4.6% of adults having moderate to severe kidney disease (14). More than 80% of the continental burden of CKD is in sub-Saharan Africa (SSA), with the highest prevalence in West Africa (15). Every year, over 500, 000 people in sub-Saharan Africa develop CKD, which causes premature death (16). In sub-Saharan Africa, the CKD burden is much greater and associated with additional risk factors like poverty, infections, a low level of health literacy, and the high cost of medical fees for screening and treatment, which collectively aggravate the risk and progression of the problem with a declining probability of survival (6, 17).

The time-to-occurrence of CKD (the time at which patients developed CKD since they were diagnosed with DM) varies across different studies as compared to the global status (3.8–12 years); the median time to develop CKD in Ethiopia is estimated as 5–8.3 years (18–22).

To address the worrisome situation of CKD incidence and its determinants among diabetic patients in Ethiopia, particularly in the Harari region, we aim to determine the incidence of CKD and identify possible factors predicting CKD among DM patients following their treatment at governmental hospitals in the Harari region of Eastern Ethiopia.

## Materials and methods

### Study design setting and period

An institution-based retrospective follow-up study was conducted among type 1 and type 2 DM patients at the governmental hospitals of the Harari region. The study was conducted at the governmental hospitals of the Harari region, which is situated in the Harari regional state and 526 km away from Addis Ababa, the capital city of Ethiopia.

The population of the Harari region was projected to be 270,000, with a 1:1 male-to-female ratio (23). These people are currently being served by two public, two private, one police, and one non-government hospital in the region. This study included all of the three governmental hospitals found in the region. These hospitals are Hiwot Fana Comprehensive Specialized University Hospital (HFCSUH), Jugal General Hospital (JGH), and Harar Federal Police Hospital (HFPH). These hospitals have diabetic care units for DM patients under their respective established chronic follow-up clinics. Currently, a total of approximately 1,632 patients with DM visit these hospitals to receive their regular follow-up care. The patients’ medical record notes from 1 September 2012 to 30 May 2022 were extracted between 1st and 31st September 2022.

### Populations and eligibility

All T1DM and T2DM patients on chronic follow-up for treatment at governmental hospitals in the Harari region and whose age is greater than or equal to 15 years were the source population, whereas all newly diagnosed T1DM and T2DM patients whose age is greater than or equal to 15 years at the governmental hospitals in the Harari region were our study population. Newly diagnosed T1DM and T2DM patients whose age is greater than or equal to 15 years were selected randomly and included in the study, whereas patients who had CKD at the start of the study, transfer-in patients, and patients with unknown date of CKD diagnosis within the follow-up period were excluded from the study.

### Sample size

The final required sample of 520 was determined via Stata software using power analysis for the Cox proportional hazard model by considering a 15.56% probability of events and 0.51 AHR of sex from previous studies (20). We assumed a 95% confidence level, 80% power, 10% withdrawal probability, and 5% for incomplete charts.

The total sample of 520 was proportionally allocated for each of the three Hospitals based on the number of DM patients who visit each hospital for follow-up care. Finally, the required number of participants was selected by simple random sampling using a computer-generated random sample from the sampling frame (the list of diabetic patients enrolled from 1 September 2012 to 30 May 2022).

### Study variables

#### Outcome variables: the incidence of CKD

*Socio-demographic factor*: Age, sex, residence, and family history of CKD.

*Clinical factors*: Proteinuria, chronic complications, glycated hemoglobin (HbA1c), low-density lipoprotein, duration of DM, total cholesterol, triglycerides, HDL-C level, acute complication, fasting blood sugar level, comorbidity, systolic blood pressure, diastolic blood pressure, types of DM, and types of treatment used were the independent variables.

### Data collection instrument and procedure

The data were collected using a data extraction format, which was adapted from the World Health Organization guidelines (24). The data extraction format contains four parts entitled with general information, baseline socio-demographic characteristics, clinical characteristics and measurements, and the outcome of interest.

Secondary data, from patient intake forms, follow-up cards, and DM registration books, as well as the electronic information databases, which are routinely recorded by the hospitals for follow-up, monitoring, and evaluation purposes, were recorded. The data collectors filled in the responses to the questions in the data extraction form. The reviewed records were identified by their medical registration number. Data were collected by four public health staff of Haramaya University, and data collection was supervised by two trained supervisors.

### Data quality control

To maintain the quality of the data, a one-day training focusing on the data extraction approach, data collection tools, and objectives of the study was given to data collectors and supervisors. Before actual data collection, a preliminary review was conducted on a 5% (25) sample size a week before starting the actual data collection, to check the adequacy of the instrument, the time required to fill the checklist of tools, and the completeness of data for charts. Necessary adjustments were made to the data abstraction format. Close follow-up and supervision were carried out during the data collection period jointly by the principal investigator and the supervisors. The collected data were reviewed and checked for completeness before data entry. Misclassification bias was attempted to be minimized by using uniform ascertainment criteria for those clinical variables having more than one diagnostic criteria.

### Operational definitions

*Diabetes Mellitus*: If DM is recorded on the patient’s medical records.

*Chronic Kidney Disease*: If CKD is recorded on DM patient medical records.

*Newly Diagnosed Diabetic Patients*: Patients who were diagnosed with diabetes between 1 September 2012 and 30 May 2022.

*Event*: CKD.

*Censored*: explained by newly diagnosed diabetic patients who had not been diagnosed with CKD until the end of the study, died, lost to follow-up, or transferred out.

*Hypertension*: If hypertension is recorded on the patient’s medical records.

*All lipid profiles*: High-density lipoprotein cholesterol (HDL-C), low-density lipoprotein cholesterol (LDL-C), triglyceride, and total cholesterol were categorized for analysis based on the Mayo Clinic and World Health Organization (WHO, 2006) guidelines.

*Proteinuria*: Urine dipstick result was used to determine urine albumin level which was reported as negative, or + 1, to +4 (25).

*Incomplete charts*: A patient chart that missed the date of DM diagnosis and the date of CKD was developed.

### Statistical analysis

The data were checked and cleaned for completeness, entered into Epi-data version 4.6, and then exported to STATA software version 17 for further data cleaning and analysis. Variables related to the lipid profile had missing data. A complete case analysis approach of missing data handling mechanisms was used. Descriptive analyzes were done in terms of mean and standard deviation or median and interquartile range for continuous variables, whereas frequency distribution and percentage were used for categorical variables, including the outcome of the study.

The cumulative incidence of CKD was calculated by taking the number of new CKD cases as the numerator and the total initial population at risk on follow-up as the denominator. Patient year at risk of developing CKD was calculated from the baseline appointment date to either the date of events or censoring. Accordingly, incidence density was computed as the number of new cases divided by patient year at risk. The outcome variables were dichotomized into CKD (Event) and censored. The Kaplan–Meier failure curve and a log-rank test were done to estimate the probability of CKD and to test the equality of failure functions among explanatory variables, respectively. Cox PH and three parametric models (Weibull, exponential, and Gompertz) were fitted to identify the predictors of CKD. The most parsimonious model was selected using the Akaike information criterion (AIC), Bayesian information criterion (BIC), and log-likelihood criterion.

**Figure 1:**
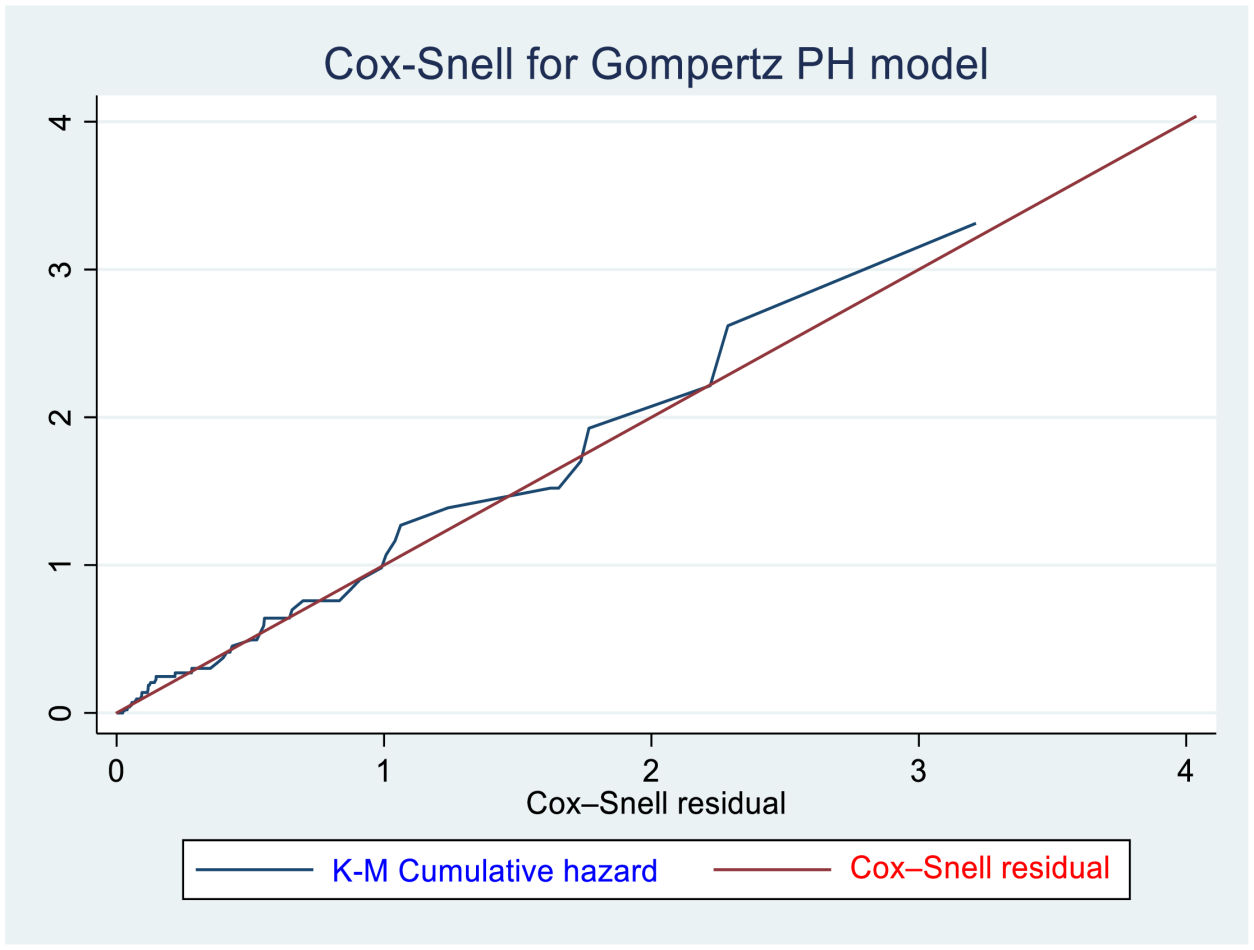
Cox–Snell residuals for Gompertz PH models of newly diagnosed T1DM and T2DM patients in the governmental hospitals in the Harari region, eastern Ethiopia from 2012 to 2022.

We used a Gompertz PH regression model to identify the predictors of CKD. The Schoenfeld residual test (both global and scaled) and graphical (log–log plot of survival) methods were used to check the proportional hazard (PH) assumption and martingale residuals to look for non-linearity of covariates with hazard rates. According to the Schoenfeld residual global test, the overall full model did not violate the proportional hazard assumption (*X*^2^ = 5.31, *p* = 0.6465) (Supplementary material S2). The presence of multicollinearity was checked by using the variance inflation factor (VIF = 2.37). A value of *p* <0.05 was used to declare statistical significance in the multivariable model, and the hazard ratio (HR) with its 95% confidence interval was computed to show the strength of the association. The goodness of fit of the model was assessed using the Cox–Snell residual technique. The cumulative hazard plot follows a straight line through the origin with slope one indicating the model is good (Figure 1).

## Results

### Baseline characteristics

Of 520 total samples, 26 (5%) patients with incomplete information were excluded, making a 95% response rate. Then, 494 DM patients were retrospectively followed from 1 September 2012 to 1 September 2022.

Of the total 494 newly diagnosed DM patients, 308 (62.35%) were men. The mean age at study initiation was 46 ± 15 years. Of the majority of DM patients, 282 (57.09%) were urban dwellers (Table 1).

**Table 1:** Incidence of CKD and baseline socio-demographic characteristics of diabetic patients.

Characteristics	Category	CKD status	
		Censored (*n* = 443)	Event (*n* = 51)
Follow-up	HFCSUH JGH FPH	200 143 100	30 12 9
Age	<60 years ≥60 years	408 35	19 32
Sex	Male Female	268 175	40 11
Residence	Urban Rural	254 189	28 23

### Clinical and follow-up characteristics

According to this study, nearly three-fourths, 375 (75.91%) of the DM patients were T2DM. Regarding acute complications, 107 (21.66%) of the patients had acute complications, of which 70 (65.42%) had diabetic ketoacidosis (DKA). Approximately 201 (40.7%) DM patients had pre-existing hypertension. Of these hypertensive patients, 177 (88.05%) of them were those with T2DM, while the remaining 24 (11.94%) patients had T1DM. The 25th and 75th percentile durations of diabetes were 3 years and 6 years, respectively, with a median duration of 4 ± 3 years. The majority of the patients had poor glycemic control (high FBS) (67.41%), total cholesterol levels less than 200 mg/dL (55.83%), and triglyceride levels less than 150 mg/dL (50.76%). Similarly, approximately 192 (48.86%) of DM patients had HgA1c levels greater than or equal to 7%, and 109 (22.24%) of patients were positive proteinuria. The majority of the patients, 356 (72.06%), received oral hypoglycemic therapy (Supplementary material S1).

### Incidence and median time of chronic kidney disease

**Figure 2:**
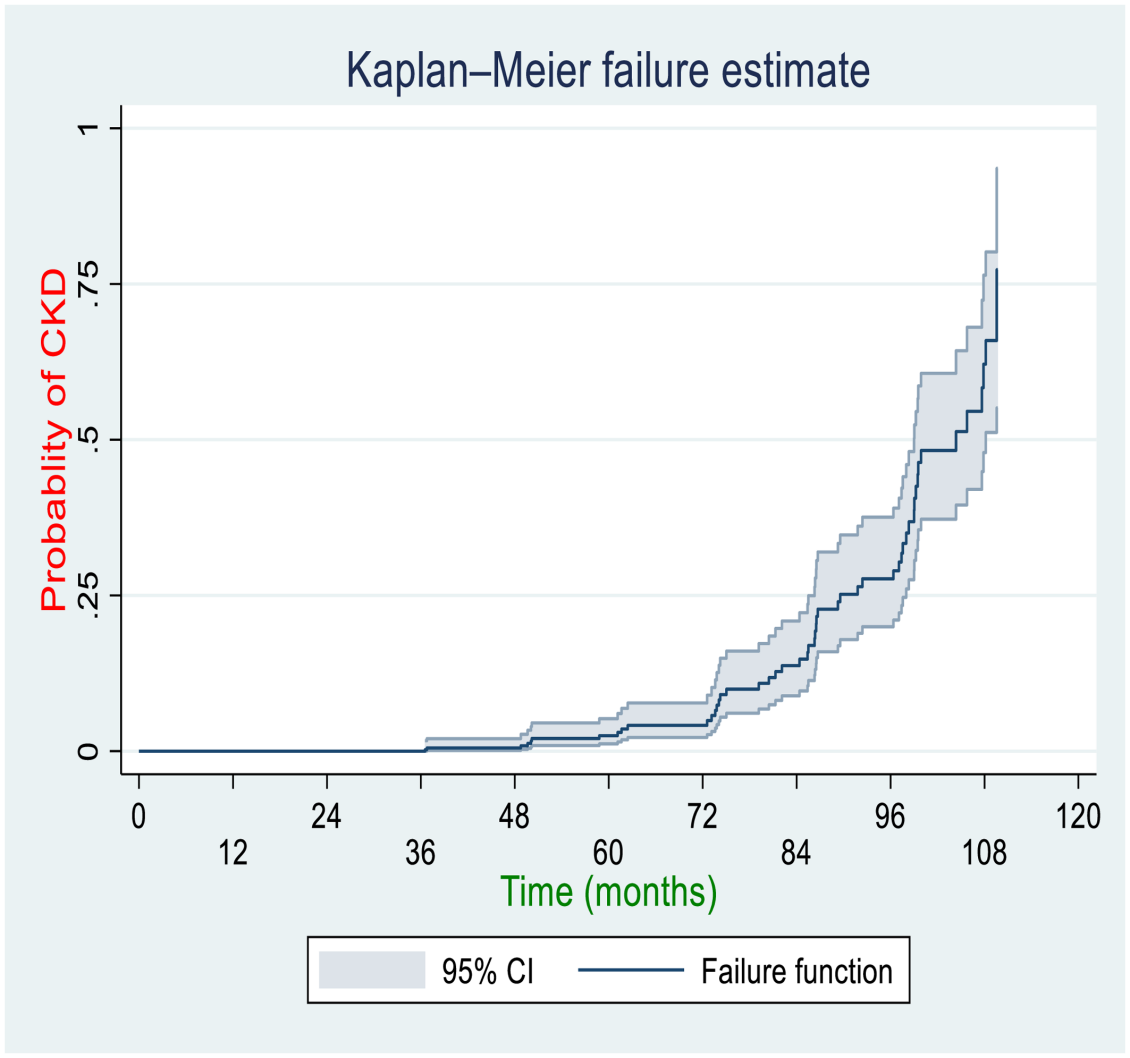
Overall Kaplan–Meier curve for the cumulative probability of CKD among diabetic patients in the governmental hospitals in the Harari region, eastern Ethiopia, 2022.

Among the total diabetic patients followed for approximately 10 years, 51(10.32%) of them with 95% CI (7.92–13.34%) developed CKD with 8.7 years of median time. The incidence density of CKD in the cohort during a cumulative 2,356 person-years of observation (PYO) was found to be 2.16 cases per 100 person-years of observation with a 95% CI of 1.64–2.84. The cumulative incidence rates (per 10,000 person-months of follow-up) of CKD at the Federal Police Hospital, Hiwot Fana Specialized University Hospital, and Jugal General Hospital were 19, 16, and 17, respectively. The Kaplan–Meier curve showed that DM patients had a higher likelihood of developing CKD over time. A large number of CKD was developed between 72 and 108 months of follow-up among DM patients. Patients’ cumulative failure estimates were 0.0024 at 36.5 months, 0.0145 at 72 months, 0.25 at 89.5 months, 0.5 at 104 months, and 0.773 at the end of the study (109.7 months) (Figure 2).

### Failure probability among covariates

**Figure 3:**
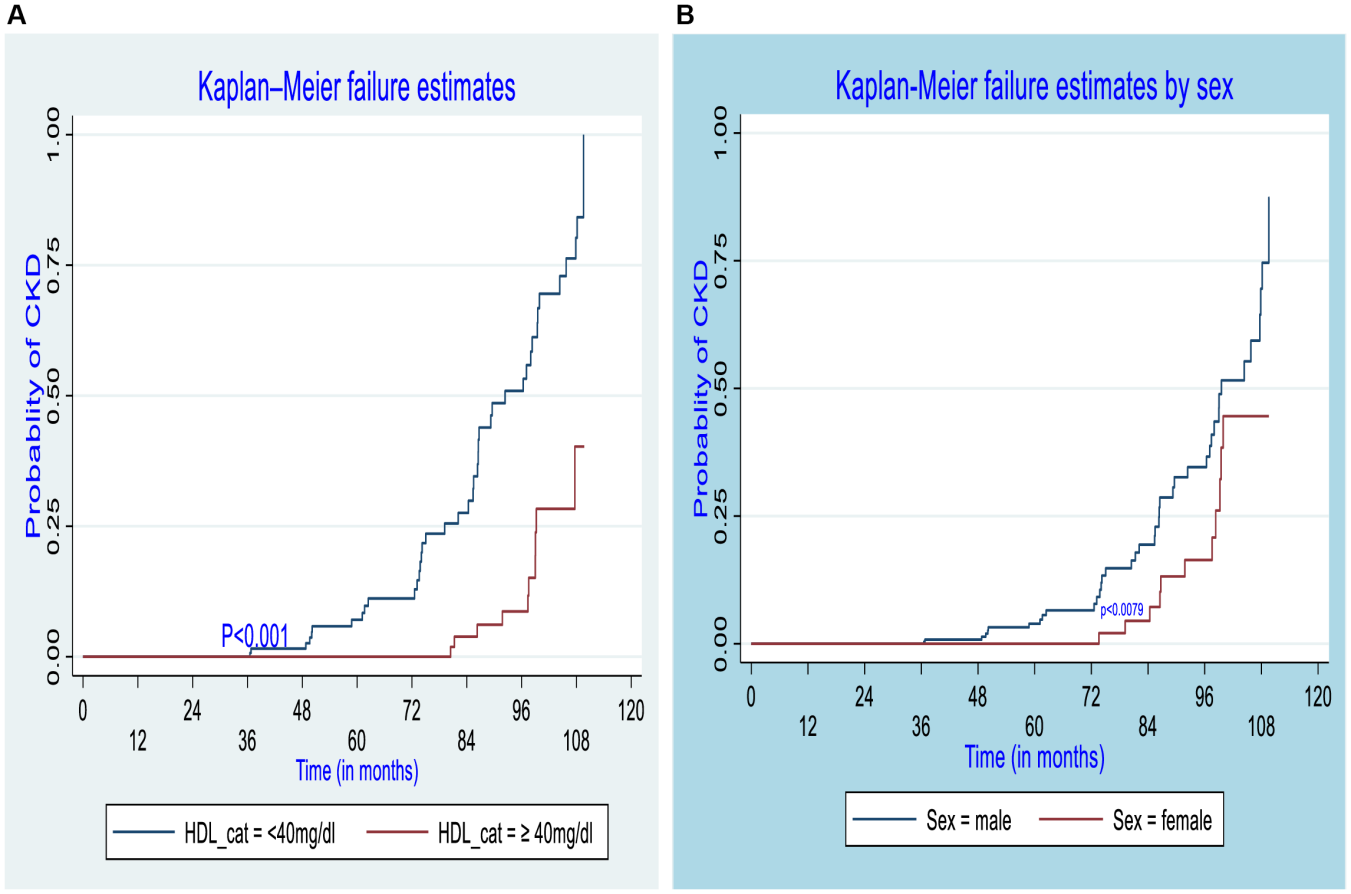
Kaplan–Meier curve for CKD estimate among different groups of diabetic patients by high-density lipoprotein **(A)** and sex **(B)** of participants in the governmental hospitals in the Harari region, eastern Ethiopia, 2022.

The log-rank test was performed to test the equality of the probability of CKD among various levels of the categorical variables. Regarding the sex of DM patients, for both male and female patients, their failure probability was equal for the first 3 years; however, in the 10th year of the follow-up, the failure probability of the male and female patients was 87.3 and 44.56%, respectively (*p* = 0.0079). Similarly, the failure probability of participants who had developed chronic complications was equal to those who did not develop chronic complications for the first 3 years; however, the overall 10-year failure probability of participants who developed chronic complications was approximately 99.9%, while that of DM patients who did not develop chronic complications was 37.32% (*p* = 0.001). On the other hand, the 10-year overall failure probability after newly diagnosis of diabetes was 40.27% for patients whose HDL-C was greater than or equal to 40 mg/dL and 99.9% for those whose HDL-C was less than 40 mg/dL (*p* = 0.001). Similarly, for those with low-density lipoprotein higher than or equal to 100 mg/dL was 99.9%, whereas for those with low-density lipoprotein less than 100 mg/dL was 41.46%(p = 0.001). Furthermore, the overall 10-year probability of developing CKD was also higher among T2DM (87.95%) as compared to T1DM (18.30%) (Figure 3).

### Predictors of CKD among DM patients

After multivariable analysis using the Gompertz PH regression model, covariates such as age, duration of DM, protein urea at baseline, and HDL-C level were found to be independent predictors for CKD among DM patients (Table 2). Accordingly, the risk of developing CKD was 3 times higher among diabetic patients aged ≥60 years as compared to those aged <60 years (AHR: 3.09, 95% CI: 1.56, 6.14). The risk of developing CKD was 3.8 times higher among DM patients with positive proteinuria as compared to their counterparts, after adjusting for other variables in the model (AHR: 3.78, 95% CI,1.80–7.91). The risk of experiencing CKD was nearly 4 times higher among diabetic patients with HDL-C less than 40 mg/dL as compared to those patients with HDL-C greater than or equal to 40 mg/dL, after adjusting for other variables in the model (AHR: 3.84, 95% CI, 1.80–8.18). Furthermore, the risk of developing CKD was 3.5 times higher among newly diagnosed DM patients with a duration greater than 5 years as compared to those patients with a duration less than or equal to 5 years (AHR: 3.53, 95% CI, 1.43–8.71).

**Table 2:** Bivariable and multivariable analysis using the Gompertz PH regression model for predictors of chronic kidney disease among DM patients in the governmental hospitals in the Harari region, from 2012 to 2022.

Variables	Category	CKD status		CHR (95% CI)	AHR (95%CI)
		Censored (*n* = 443)	Event (*n* = 51)		
Age	≥60 years <60 years	35 408	32 19	13.25(7.51–23.38) 1	3.09(1.56–6.14)** 1
Sex	Male Female	268 175	40 11	2.11(1.08–4.11) 1	1.005(0.48–2.08) 1
Duration of DM	>5 years ≤5 years	159 284	44 7	9.90(4.46–21.98) 1	3.53(1.43–8.71)* 1
Proteinuria	Positive Negative	70 373	39 12	14.32(7.49–27.37) 1	3.78(1.80–7.91)** 1
Total cholesterol	≥200 mg/dL <200 mg/dL	142 210	36 15	2.88 (1.57–5.26) 1	1.59(0.77–3.26) 1
HDL	<40 mg/dL ≥ 40 mg/dL	143 199	41 10	4.81 (2.41–9.61) 1	3.84(1.80–8.18)** 1
Comorbidity	Yes No	187 256	44 7	7.68 (3.46–17.05) 1	2.11 (0.85–5.24) 1
Types of DM	Type 2DM Type 1DM	329 114	46 5	2.98(1.18–7.50) 1	0.98 (0.34–2.85) 1
Fasting blood sugar	>150 mg/dL ≤150 mg/dL	293 150	40 11	1.74 (0.89–3.39) 1	1.22(0.61–2.42) 1

Value of *p* (* < 0.05, ** < 0.01), HDL-C, high-density lipoprotein cholesterol; gamma = 0.006,1 shows the reference group of different variables.

## Discussion

### Aim of study

This study aimed to determine the incidence and possible predictors of CKD among DM patients following their treatment at governmental hospitals in the Harari region of eastern Ethiopia.

### Comparison with literature

#### Incidence of CKD

In our study, 51(10.32%) of the DM patients had developed CKD, with an incidence density of 2.16 per 100 person-years (PY) of observation. In this study, the median occurrence of CKD was 8.7 years. A total of four factors, namely age, proteinuria, the duration of DM, and HDL-C, were found to be significantly associated with the risk of CKD.

The cumulative incidence of CKD in this study aligns with findings from studies done elsewhere in Ethiopia(10.8%) (22), Korea (12.1%) (26), and Spain (10.23%) (27); however, it was lower than studies conducted at St. Paul’s Hospital Addis Ababa, Ethiopia (14.25%) (18), Jimma University Medical Center, Ethiopia (15.56%) (20), and the United Kingdom (29%) (28).

The observed discrepancy may be because participants in the study at Jimma Medical Center were only newly diagnosed with diabetes at least 5 years before the date of data extraction, which likely increased the duration of the DM. Participants in previous studies at St. Paul’s Hospital Addis Ababa were urban dwellers; their lifestyle may have distorted the metabolic system and contributed to renal impairment. However, in this study, 43% of participants were from rural areas. On the other hand, the study design used in the United Kingdom was a prospective follow-up study. To calculate the patient’s estimated glomerular filtration rate using the serum creatinine level, venous blood was directly collected from the patient. The estimated glomerular filtration rate was used to evaluate the patient’s status, and unfortunately, even those who did not exhibit any symptoms of CKD may be labeled as CKD if their e-GFR is below 60 mL/min/1.73 m^2^. However, in this study, participants were only considered to have CKD if a diagnosis of CKD was present on their medical records. This might underestimate the incidence of CKD in diabetic patients.

#### CKD and median time

The median failure time was 8.7 years, which is higher than the study conducted at Amhara region referral hospitals(5 years) (22), St. Paul’s Hospital Addis Ababa, Ethiopia(6.7 years) (18), and Jimma University Medical Center, Ethiopia (5.8 years) (20). This difference might be due to the early diagnosis and treatments of participants in this study. However, it is shorter than the finding of a study conducted in the United Kingdom (12 years) (28). This variation may be caused by the difference in duration of the study and participants selected in the United Kingdom were residents of civilized cities, where it is predicted that their knowledge and health-seeking behavior are expected to be better, which could allow them to use better self-care practices.

#### CKD and old age

Our finding showed a significant association between older age and CKD. This finding is congruent with a study conducted at St. Paul’s Hospital in Addis Ababa, Ethiopia (18), Taiwan (29), and Italy (30), this is because as age increases, there is a progressive loss of nephrons, decreased renal blood flow, and diminished renal function in older DM patients than younger ones which lead to CKD (31). After the age of 30 years, GFR progressively declines at an average rate of 8 mL/min/1.73 m2 per decade which might be due to structural and functional changes in the kidney in older patients (32). The other reason might be older age is associated with risk factors of CKD such as cardiovascular diseases and obesity (33). Therefore, screening for CKD in an old age group is an important strategy to take an appropriate intervention.

#### CKD and positive proteinuria

In this study, proteinuria was positively associated with CKD. The risks of developing CKD were higher in diabetics with positive proteinuria than in those with negative proteinuria. In addition to being a sign of kidney impairment, proteinuria also causes progressive kidney injury (34). Proteinuria is caused by either functional or structural alterations of glomerular filtration or proximal tubule dysfunction (35). Renal tubular cells are injured when urine protein levels rise due to glomerular capillary wall damage or a decrease in tubular protein reabsorption. When renal tubules are exposed to urinary proteins, it results in fibrosis and interstitial inflammation (34).

This finding is in line with the findings of a study conducted at Jimma University Medical Center (20) and the University of Gondar Referral Hospital, Ethiopia (36).

#### CKD and duration of DM

The duration of the patients who stayed with DM was significantly associated with the development of CKD among diabetic patients. This finding is in line with the systematic review and meta-analysis conducted in Ethiopia (37), a study conducted at the University of Gondar Comprehensive Specialized Hospital, Northwest Ethiopia (21), and a study conducted in Italy (38). Over time, kidney blood vessels and nephrons can be harmed by diabetes-related elevated blood sugar, inhibiting their ability to function as they should (39). According to a study on risks of rapid decline of renal function in patients with type 2 diabetes, women and men with diabetes experienced eGFR declines of 2.1 and 2.7 mL/min per 1.73 m2 each year, respectively (40). This shows that improving the way diabetes care is provided before CKD develops may prevent the occurrence and progression of early diabetic CKD.

#### CKD and HDL cholesterol

In this study, higher levels of HDL cholesterol (≥ 40 mg/dL) were associated with a lower risk for CKD. This result is consistent with other studies done in different parts of Ethiopia, at St. Paul’s Hospital Addis Ababa (18), Jimma University Medical Center (20), and University of Gondar Referral Hospital (36) and studies conducted in Italy (41). This might be due to the fact that, biologically, HDL-C reduces the accumulation of fats and atherosclerosis within the arterial wall by transporting lipids away from the artery wall to the liver. In addition, it protects the inner wall of the arteries from damage so this reduces the risk of vascular complications of DM including CKD (42). HDL-C, in contrast to LDL, has anti-atherogenic properties that include reverse cholesterol transport, anti-oxidant and anti-inflammatory properties, and maintenance of endothelial function (43). This could be also achieved through medication like SGLT2 inhibitors, which enhance vascular tone, elasticity, and contractility by lowering inflammation, oxidative stress, insulin signaling, and endothelial cell proliferation (44). In addition to the above-identified risk factors, there might be rare genetic diseases that can have a significant impact on kidney function, leading to various kidney disorders. These can be manifested in various forms, including inherited renal disorders, cystic kidney diseases, glomerulopathies, and tubulopathies, which can be identified through genetic testing. These conditions are often caused by mutations in specific genes that disrupt normal kidney development, function, or metabolism. Their prevalence is not clearly stated in Ethiopia.

### Clinical relevance

The clinical importance of this study was to provide information on the incidence and predictors of CKD and enhance healthcare professionals to prevent its further progression. The public health importance of this study was to reduce the economic loss for the treatment of CKD and its complications through early treatment and follow-up of older DM patients with advanced disease status at baseline. Moreover, it also helps to reduce years of life lost associated with CKD and its complications.

### Strength and limitations

#### Strength of the study

The study was conducted for a 10-year follow-up that helps to show the long-term impact of DM on CKD. The multicenter nature of the study could allow for reflecting on the regional burden of CKD and making generalizations.

#### Limitations of the study

The limitation of this study was the use of secondary data that resulted in incompleteness, which could underestimate the findings and reduce the statistical power of the study. Some potentially important predictors such as educational status, occupation, substance use, self-care practice, habitual use of antipain, and BMI were not available, which prevented us from studying their association with CKD. Moreover, this study relied on physicians’ judgments to define or diagnose CKD, which might not be sufficiently reliable on its own.

## Conclusion

In this study, we found that one in 10 diabetic patients had developed CKD within 10 years of the DM diagnosis. Advanced age, longer duration of diabetes, lower baseline HDL-C level, and proteinuria had increased the hazards of developing CKD. We recommend a more targeted follow-up of older adults patients with advanced disease status at baseline for optimal control of DM in order to prevent its furthering to CKD.

## Data availability statement

The raw data supporting the conclusions of this article will be made available by the authors, without undue reservation.

## Ethics statement

The studies involving humans were approved by Institutional Health Research Ethics Review Committee of the Haramaya University College of Health and Medical Sciences. The studies were conducted in accordance with the local legislation and institutional requirements. The ethics committee/institutional review board waived the requirement of written informed consent for participation from the participants or the participants’ legal guardians/next of kin because due to retrospective nature of the study.

## Author contributions

AC: Conceptualization, Formal analysis, Methodology, Writing – original draft, Writing – review & editing. DE: Data curation, Writing – review & editing. LR: Conceptualization, Data curation, Supervision, Writing – review & editing. TG: Conceptualization, Data curation, Supervision, Writing – review & editing.

## Figures and Tables

**Table:** 

ADA	American diabetics association
AHR	Adjusted hazard ratio
BMI	Body mass index
CHR	Crude hazard ratio
CKD	Chronic kidney disease
DBP	Diastolic blood pressure
DM	Diabetes mellitus
DN	Diabetic nephropathy
e-GFR	Estimated glomerular filtration rate
FPH	Federal Police Hospital
HFCSUH	Hiwot Fana Comprehensive Specialized University Hospital
HDL-C	High-density lipoprotein cholesterol
HbA1c	Hemoglobin a1c
IDF	International Diabetic Federation
JGH	Jugal General Hospital
LDL-C	Low-density lipoprotein cholesterol
MOH	Ministry of Health
PH	Proportional hazard
SBP	Systolic blood pressure
T1DM	Type one diabetes mellitus
T2DM	Type two diabetes mellitus
WHO	World Health Organization
