# Supplementary material for: Incidence and predictors of chronic kidney disease among patients with diabetes treated at governmental hospitals of Harari Region, eastern Ethiopia, 2022
Source: Front Public Health. 2024 Jan 5;11:1290554. doi: 10.3389/fpubh.2023.1290554 (PMC10797702; doi:10.3389/fpubh.2023.1290554)
Supplement: Supplementary file 1 [file Table_1.DOCX]

*Additional files- 1: CKD incidence and baseline clinical and follow-up characteristics of diabetic patients at governmental hospitals of Harari region, Eastern Ethiopia from 2012 to 2022 (N=494).*

| **Variables** | **Category** | **CKD Status** | |
| --- | --- | --- | --- |
|  |  | **Censored (n=443)** | **Event (n=51)** |
| Types of DM | Type 1DM | 114 | 5 |
|  | Type 2DM | 329 | 46 |
| Family history of CKD | No | 369 | 42 |
|  | Yes | 74 | 9 |
| Acute Complication | No | 363 | 24 |
|  | Yes | 80 | 27 |
| List of Acute complications (if acute complication yes) | DKA | 64 | 6 |
|  | HHNS | 16 | 21 |
| Chronic Complication | No | 382 | 15 |
|  | Yes | 61 | 36 |
| List of Chronic Complications (if chronic complication yes) | Diabetic retinopathy | 11 | 13 |
|  | Diabetic foot ulcer | 9 | 13 |
|  | Diabetic Neuropathy | 24 | 4 |
|  | Diabetic nephropathy | 17 | 6 |
| Comorbidity | No | 256 | 7 |
|  | Yes | 187 | 44 |
| List of Comorbidity | Hypertension(yes) | 160 | 41 |
|  | Stroke(yes) | 49 | 8 |
|  | Myocardial infarction (yes) | 3 | 1 |
|  | Dyslipidemia | 21 | 2 |
|  | Other comorbid | 3 |  |
| Duration of DM (in years) | ≤5 | 284 | 7 |
|  | >5 | 159 | 44 |
| Systolic blood pressure (mmHg) | Mean(SD) | 131.5(17) | 138.2(29) |
| Diastolic blood pressure (mmHg) | Mean (SD) | 81(10) | 82(16.3) |
| Fasting blood sugar (mg/dl) | ≤150mg/dl | 150 | 11 |
|  | >150mg/dl | 293 | 40 |
| Total cholesterol(mg/dl) | <200mg/dl | 210 | 15 |
|  | ≥200mg/dl | 142 | 36 |
| Triglycerides(mg/dl) | <150mg/dl | 190 | 11 |
|  | ≥150mg/dl | 155 | 40 |
| HDL (mg/dl) | <40 mg/dl | 143 | 41 |
|  | ≥ 40mg/dl | 199 | 10 |
| LDL (mg/dl) | <100mg/dl | 239 | 21 |
|  | ≥ 100 mg/dl | 107 | 30 |
| HgA1C | <7% | 184 | 17 |
|  | ≥ 7% | 158 | 34 |
| Proteinuria | Negative | 373 | 12 |
|  | Positive | 70 | 39 |
| Types of Treatment | OHA | 310 | 38 |
|  | Insulin | 104 | 8 |
|  | Mixed | 21 | 5 |

Additional files-2:Test of proportional hazard assumption based on Schoenfeld residuals test for the covariates

| **Variable** | **Rho** | **Chi-square** | **Df** | **P-value** |
| --- | --- | --- | --- | --- |
| Proteinuria | -0.13892 | 1.02 | 1 | 0.3132 |
| HDL | 0.10049 | 0.65 | 1 | 0.4205 |
| Duration | -0.26593 | 1.03 | 1 | 0.3022 |
| Age | -0.01001 | 0.01 | 1 | 0.9360 |
| Comorbidity | -0.06706 | 0.25 | 1 | 0.6154 |
| Types of DM | -0.00307 | 0.00 | 1 | 0.9865 |
| FBS | 0.14222 | 1.11 | 1 | 0.2930 |
| Total cholesterol | 0.04936 | 0.18 | 1 | 0.6733 |
| Sex | -0.22887 | 1.06 | 1 | 0.3011 |
| Global test |  | 5.31 | 9 | 0.6564 |
